# Pilot study to evaluate a tailored text message intervention for pregnant smokers (MiQuit): study protocol for a randomised controlled trial

**DOI:** 10.1186/s13063-014-0546-4

**Published:** 2015-01-27

**Authors:** Sue Cooper, Katharine Foster, Felix Naughton, Jo Leonardi-Bee, Stephen Sutton, Michael Ussher, Matthew Leighton, Alan Montgomery, Steve Parrott, Tim Coleman

**Affiliations:** Division of Primary Care, University of Nottingham, University Park, Nottingham, NG7 2RD UK; Department of Public Health and Primary Care, Institute of Public Health, University of Cambridge, Forvie Site, Robinson Way, Cambridge, CB2 0SR UK; Division of Epidemiology and Public Health, University of Nottingham, Clinical Sciences Building, City Hospital, Hucknall Road, Nottingham, NG5 1PB UK; Population Health Research Institute, Hunter Wing, St George’s, University of London, Cranmer Terrace, London, SW17 0RE UK; Nottingham Clinical Trials Unit, University of Nottingham, Nottingham Health Science Partners, C Floor, Queen’s Medical Centre, Nottingham, NG7 2UH UK; Department of Health Sciences, Seebohm Rowntree Building, University of York, Heslington, York, YO10 5DD UK

**Keywords:** Smoking cessation, Pregnancy, Self-help, Randomised controlled trial, Protocol

## Abstract

**Background:**

Smoking in pregnancy is a public health problem. Self-help smoking cessation support can help pregnant women to stop smoking, but the effects of delivering this kind of support via SMS text message are not known. A previous randomised controlled trial (RCT) demonstrated the feasibility and acceptability of providing such support to pregnant smokers using an automated, tailored text message intervention called MiQuit. This larger RCT will estimate key parameters for and will test the feasibility of delivering a major trial run within the United Kingdom National Health Service settings aimed at providing definitive evidence on the utility of MiQuit for helping pregnant smokers to stop.

**Methods/Design:**

This will be a multi-centre, parallel group RCT. Participants are being identified in 16 English antenatal care settings and must be >16 years old, pregnant, <25 weeks gestation, smoke >1 daily cigarette, have smoked >5 daily cigarettes before pregnancy, and able to understand texts in English. After consenting and the collection of baseline data, participants are randomised to control or intervention groups in a 1:1 ratio; randomisation is stratified by trial site and gestation and employs computer-generated pseudo-random code using random permuted blocks of randomly varying size, and held on a secure server. All participants receive a National Health Service (NHS) leaflet aimed at helping them to stop smoking. Intervention group women also receive the 12-week MiQuit programme of tailored, supportive, interactive text message, self-help cessation support. Women are followed up by telephone 4 weeks after randomisation and at 36 weeks gestation. The study aims to recruit 400 women, and with this sample we will be able to estimate trial centres’ recruitment rates to within +/−1% (margin of error = half width of 95% confidence interval); individual trial groups’ ascertainment of rates for smoking outcomes between 4 weeks after randomisation until approximately 36 weeks gestation to within +/−4%, and across both groups, the combined cessation rate at 36 weeks +/−3%.

**Discussion:**

Pilot trial completion will provide data to facilitate planning for a definitive trial investigating whether MiQuit works for smoking cessation in pregnancy.

**Trial registration:**

ClinicalTrials.gov NCT02043509 Registered 14 January 2014.

## Background

Epidemiological evidence indicates that smoking during pregnancy increases the risk of many pregnancy-related complications including low birth weight, preterm birth, spontaneous abortions, stillbirth, ectopic pregnancy and neonatal death and is associated with many adverse postnatal problems including impaired lung function and Sudden Infant Death Syndrome [1]. Taking into account that between one in four [2] and one in eight [3] women are estimated to smoke during pregnancy, the consequences of prenatal smoking come at a great financial cost to the health service.

A review of smoking cessation interventions for pregnant smokers indicates that such interventions can be effective at increasing cessation during pregnancy, although the increase is fairly modest [4]. Although smoking cessation support is offered to pregnant smokers through the NHS Stop Smoking Services in England, uptake rates are fairly low [5,6]. While it is important to explore ways of increasing the uptake of face-to-face specialist support among this population, it is equally important to explore the potential of alternative forms of support.

One such alternative is self-help. A recent review of the literature indicates that self-help interventions for pregnant smokers almost double the chances of quitting compared with usual care [7]. Furthermore, self-help has been identified as being of high interest to pregnant smokers interested in quitting smoking [8]. It is also generally of low cost and, potentially, has wide reach. One promising mode of delivery for self-help support is through mobile phone text messages. A recent large trial of a text message smoking cessation intervention for non-pregnant smokers found it doubled cessation rates compared with a no-intervention control group [9]. Regarding its potential for pregnant smokers, mobile phone ownership is high across the social class spectrum in the UK, and text messaging is particularly popular among those under 45 years of age [10].

A pilot trial of a tailored self-help intervention developed for pregnant smokers in Cambridge (called MiQuit) found that delivering support by text message to pregnant smokers was feasible and also acceptable for recipients [11]. Furthermore, the MiQuit intervention increased the likelihood of women setting a quit date and increased women’s harm beliefs (related to smoking) and confidence in their ability and determination to quit. Although this trial was small (n = 207), it provided an estimate for MiQuit’s efficacy, albeit with wide 95% confidence intervals, which suggests that this intervention could be a valuable addition to standard NHS support for smoking cessation in pregnancy. At 12 weeks after randomisation, 12.5% of women who used MiQuit reported not having smoked in the previous 7 days, which was validated using salivary cotinine assessment, compared with only 7.8% in the control group; if MiQuit could have such an impact in routine NHS care, its low cost would make it highly cost-effective.

Before MiQuit could be used in routine care, a definitive randomised controlled trial would need to prove its effectiveness, so obtaining further evidence concerning its efficacy is appropriate. However, such a trial would need to be very large and expensive and, currently, there is insufficient information to justify conducting this. For example, it is not known if cessation encouraged by MiQuit, in the pilot trial, which was measured, at 12 weeks after randomisation (around mid-pregnancy), persists beyond this point. However, sustained cessation throughout pregnancy would be required before one could expect this potentially-useful intervention to have a meaningful impact on fetal health. Also, there is only very limited information with which one could plan a definitive trial such that this made efficient use of research resources; for example, likely recruitment and follow-up rates need determining. Consequently, we propose a pilot trial, which will investigate whether or not it is possible to evaluate MiQuit in a multi-centre RCT located within the NHS research infrastructure, taking full advantage of appropriate NHS research network resources. This study will provide estimates for the following parameters, which will be of crucial importance when deciding upon resources required for a definitive trial: the range of likely recruitment rates in different centres; quit rates amongst trial participants; feasibility of assessing smoking status of trial participants in later pregnancy; ascertainment rates obtained; and the likely effect of MiQuit when women are offered this in NHS settings. Findings from the pilot trial will permit estimation of the likely impact of MiQuit being introduced into routine clinical practice.

### Aims and objectives

The aim of this trial is to estimate the likely impact of the MiQuit text message-based smoking cessation intervention for pregnant smokers and to establish robust estimates for key parameters, which would be required in order to design a definitive trial of this intervention.

### Primary objective

The primary objective of this study is to estimate the key parameters required in order to design a definitive trial testing the efficacy of MiQuit, including the following:

1. Recruitment rates within pregnant smokers attending hospital recruitment centres
2. For individual trial groups, rates of ascertainment of validated smoking cessation between 4 weeks after randomisation until late pregnancy (approximately 36 weeks)
3. For both trial groups, the combined quit rate at 36 weeks (cessation as defined above).

### Secondary objectives

The secondary objectives are as follows:

1. To estimate and model the likely effectiveness and cost effectiveness of MiQuit compared with usual care including a generic smoking cessation information leaflet.
2. To document participants’ use of MiQuit interactive features and of NHS cessation support.
3. To assess the effect of MiQuit on social cognitive determinants of quitting smoking.
4. To explore participant views and experiences of using MiQuit.
5. To explore research staff views and experiences of the MiQuit pilot trial.

## Methods/Design

### Trial design

This study is a multicentre, two-arm, parallel group, single blind, randomised controlled trial testing the MiQuit text message smoking cessation support system in pregnant women.

### Study setting

Participants will be recruited from hospital antenatal clinics in England.

### Eligibility criteria

To be eligible for the trial participants must be i) pregnant and less than 25 weeks gestation, ii) have smoked at least 5 cigarettes per day pre-pregnancy and have continued to smoke at least 1 cigarette on a typical day during pregnancy, iii) aged 16 or over, iv) agree to accept information to assist cessation, v) have their own or have primary use of a mobile phone, vi) be familiar with sending and receiving text messages, vii) be able to understand written English (text messages are in English only) and consent issues explained in English, and viii) be able to give informed consent.

They should not already be enrolled in another text service to assist smoking cessation.

### Intervention

MiQuit is an automated responsive text message support programme lasting 12 weeks, which provides tailored smoking cessation support and advice to the participant’s mobile phone. The MiQuit system provides advice and support content that is tailored to 12 characteristics of the individual at baseline and is tailored to changes in smoking status collected at two time points during the 12-week programme. In addition, participants can text in a quit date and receive additional support tailored around this date. Participants can also increase or decrease the frequency of support they receive at any time by texting the keywords MORE or LESS. The support includes motivational messages, advice about preparing for a quit attempt, how to manage cravings and withdrawal, dealing with trigger situations, information about how smoking affects babies and general encouragement. Participants will receive between 70 and 145 texts over 12 weeks (0.8 to 1.7 texts per day), dependent upon how they use the system. They also will have the ability to request instant support text messages on demand or distraction messages by texting one of three keywords to the MiQuit system. Any text message replies to MiQuit made by participants will be charged at the usual network rate or included in their text allowance, but texts they received from MiQuit are free. All likely costs to participants will be made clear in the participant information sheet (PIS).

In addition to the MiQuit text message intervention, intervention group participants will receive a standard NHS leaflet giving some information and advice on stopping smoking and usual NHS antenatal care.

### Control

Control group participants will receive the same NHS leaflet giving some information and advice on stopping smoking in addition to usual NHS antenatal care.

### Adherence

Participants allocated to receive the MiQuit intervention will have an adherence check performed at the late pregnancy/postnatal follow-up stage, at 36 weeks gestation (visit 3). At this stage participants will be asked basic questions about their use of the texts sent.

If they wish, participants in both groups will be able to access additional support to help them stop smoking; they will all be asked about any use of support at each contact.

### Outcomes

There is no single primary outcome for this pilot trial, which deals primarily with feasibility issues.

### Feasibility outcomes

Feasibility outcomes include the following:

1. Recruitment rate of participants from pregnant smokers attending trial centres.
2. For individual trial groups, proportions of participants with validated smoking cessation data collected between 4 weeks after randomisation until late pregnancy (at approximately 36 weeks gestation).
3. For both trial groups combined, collection of validated smoking cessation data, as above, measured in late pregnancy (approximately 36 weeks gestation).

### Smoking outcomes

Smoking outcomes are described below:

1. Proportion of participants with self-reported, continuous abstinence from smoking from 4 weeks after randomisation until follow-up in late pregnancy (approximately 36 weeks gestation), *validated* by exhaled CO and/or saliva cotinine estimation; there will be no ‘grace period’ [12]. Participants who report smoking no more than five cigarettes in total between the start of their quit attempt (within 4 weeks of randomisation) and late pregnancy will be considered to have quit smoking [13].
  (Note: Participants who cannot be contacted at 4 weeks after randomisation, but who, in late pregnancy, report cessation as outlined above, will be counted as positive for the late pregnancy cessation outcome).
  NB: We anticipate the above outcome being a *primary outcome* for any definitive trial that follows this study.
2. Proportion of participants with self-reported abstinence from smoking from 4 weeks after randomisation until late pregnancy (at approximately 36 weeks gestation).
3. Proportion of participants with self-reported and validated 7 day point prevalence cessation measured in late pregnancy (approximately 36 weeks gestation).
4. Proportion of participants with self-reported 7-day point-prevalence cessation at 4 weeks after randomisation.
5. Number of 24 hour quit attempts reported in late pregnancy (approximately 36 weeks gestation).

### Other outcomes

Other outcomes include the following:

1. Use of NHS cessation support.
2. Social cognitive variables such as health beliefs and outcome expectancies.
3. Process variables such as number of contacts with MiQuit for tailoring of content and increase or decrease requests.

### Assignment of interventions: allocation and blinding

After entering all baseline data onto a secure online database, research midwives or central staff will randomise participants via this database; however, these staff will be unaware of the participant’s allocated group. Randomisation will use a computer-generated pseudo-random code with random permuted blocks of randomly varying size, created by the Nottingham Clinical Trials Unit and held on a secure server. The randomisation will be stratified by site and gestation (<16 weeks versus ≥16 weeks). The database will then send an email to inform unblinded central administrators who are not involved in participant follow-up of the allocation; they will then send participants an information pack, which will provide further information on participant allocation. Local hospital staff and central staff involved in follow-up will remain blind to treatment allocation as far as possible; however, as participants are not blinded it is possible that they may unintentionally reveal their allocation during follow-up. In addition, staff carrying out the final follow-up calls will need to ask participants questions about the intervention and so will become unblinded towards the end of this call. There should be no other reasons why unblinding is needed. Researchers carrying out the analyses will remain blind. Further details are provided in the data collection sections.

### Recruitment

Women will be recruited as they attend hospitals for antenatal screening (ultrasound) appointments using one or both of two different methods, depending on the locally-available resources in participating Acute Trusts. For both recruitment methods, all pregnant women attending clinics will be asked to complete a brief questionnaire, which identifies smokers and asks those who would like to receive self-help cessation support in the context of a research study to provide contact details. A PIS will be attached to the questionnaire. Questionnaires will be distributed by a member of the patient’s usual care team (for example, receptionist or midwife); information about the trial will be displayed in relevant clinical areas and adverts in clinic environments may also be used to aid recruitment. The two methods of recruitment are described as follows:

1. *Via trial co-ordinating centre staff*: A trust staff member will oversee daily collation of questionnaires and interested women’s contact details at each recruiting site, sending these to a trial office by fax or post. A member of the study team, who has been appropriately trained, would then telephone potentially-interested women and ensure that all aspects pertaining to study participation are verbally-addressed prior to obtaining consent.
2. *Via staff in Acute Trust*: This method of recruitment will only be used in trial centres in which Comprehensive Local Research Network (CLRN) research midwives are available. Consent will, in the main, be obtained in person by a CLRN research midwife (RM); however, should the patient need to leave before consent can be obtained, a member of the team may call and consent over the telephone as for method one. A trust staff member or the RM working within these recruiting sites will oversee collation of questionnaires and the RM will follow up face-to-face those respondents who are interested in participating, usually while they are still in clinic. The RM will offer trial enrolment to all eligible, interested women ensuring that relevant aspects of trial procedures are fully explained prior to obtaining written informed consent. Those women who are not able to wait will be asked (where possible) for a convenient time to contact by telephone where the process will be fully explained and consent provided verbally.

This trial will, therefore, investigate the feasibility of the two recruitment methods and will also provide an estimate of CLRN resources that might be required for a definitive study using the second recruitment method. A variety of NHS Trusts will be used as recruitment sites to encourage diversity of recruitment and increase generalisability. Reasons why potential participants are not recruited will be collected and reported.

### Withdrawal of participants from intervention or assessments

Participants may be withdrawn from the trial either at their own request or at the discretion of the Investigator. Participants who experience miscarriage or fetal death will be advised to withdraw from the trial.

Individuals in the MiQuit arm may cancel text messages by sending a ‘STOP’ text message at any time during the programme’s duration. However, unless they specifically withdraw, they will remain in the trial as participants.

Any participant who has already participated in the study may not re-enter at a later date.

### Participant timeline and data collection

#### Pre-Screen

All potential participants will be identified via a brief questionnaire, which will be attached to a participant information sheet.

*Recruitment method one*: For recruitment by this method a member of the Co-ordinating centre study team will complete and sign the consent form noting that the consent was taken by telephone.

*Recruitment method two*: This will cover the details as for method one, except that the participant can provide either written or verbal informed consent; an RM working within the participating acute trust will explain all aspects of the study and will counter-sign the consent form in both cases.

All participants will be asked to give written informed consent for the collection of an exhaled breath and/or saliva sample if they report a quit at the week 36 follow-up. They will be made aware that the provision of samples will only be required if a quit is reported at week 36 of their pregnancy and that this may involve a visit to the participant’s home by the research midwife. If the participant is not happy to be visited at home but is happy to provide a saliva sample, postal sample kits are available. Carbon monoxide levels in exhaled breath samples can only be measured in person during a home visit.

Participants will also be given the option at the beginning of the study to consent to be contacted by the study team to discuss whether they would be interested in being interviewed as part of an intervention process evaluation. They will not be consenting to the interview at the start of the study.

All participants will be provided with clear information about how to withdraw their consent via Freepost postcard (provided to all participants), text, email, or telephone. In a similar trial in which we provided similar methods to facilitate informed consent, fewer than 60 participants (from >2,500) withdrew consent after enrolment [14].

**Figure 1:**
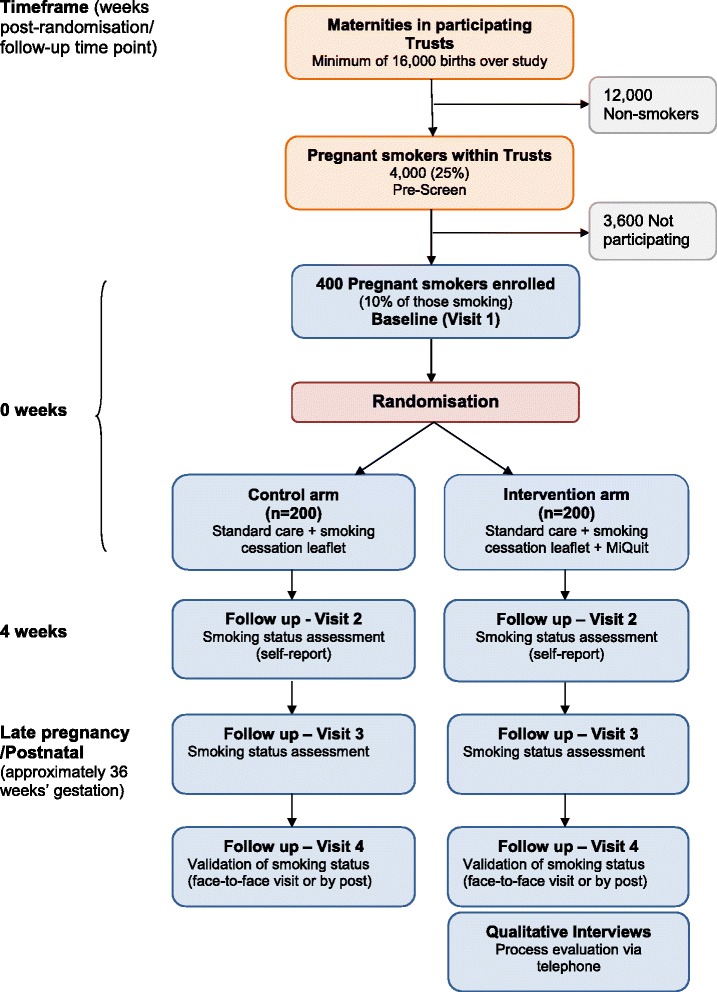
**Participant flowchart: study design and the timeframe for follow-up assessments.**

Table 1 describes the participant assessments at each stage. Figure 1 gives an overview of the study design and measurement timepoints.

**Table 1:** **Participant assessments at each time point**

**Participants Assessments**	**Pre-screen**	**Visit 1: Baseline**	**Randomisation**	**Visit 2: 4 week Follow-up**	**Visit 3: Late Pregnancy/Postnatal Follow-up**	**Visit 4: Validation of smoking status**
Trial Unit (PS)	**X**					
Acute Unit (PS)	**X**					
Consent^a,b^	**X**					**X**
Visit Dates^c^		**X**		**X**	**X**	**X**
Demographics/Education		**X**				
Smoking Behaviour 1		**X**				
Smoking Behaviour 2		**X**				
Smoking Behaviour 3						**X**
Smoking Beliefs		**X**			**X**	
Pregnancy		**X**				
Relationship Status		**X**				
Eligibility Criteria Check		**X**				
EQ-5D-3 L^d^		**X**		**X**	**X**	
Allocation - MiQuit/Control			**X**			
Abstinence Check				**X**		
Stop Smoking Strategy/Quit attempts					**X**	
Attitude to Intervention (MiQuit only)^e^					**X**	
Assessment of Tobacco Exposure^f^						**X**

^a^Telephone and written consent will be performed at Pre-screening.

^b^Written consent for cotinine sample collection will be performed at V4 for those who provided telephone consent at pre-screening.

^c^Telephone/face to face visit will be performed at V1 (only) and face to face/postal visit will be performed at V4 (only). Visits 2 and 3 will be telephone, with questionnaires sent to any who cannot be contacted.

^d^EQ-5D-3 L - European Quality of life Five Dimension, Three Levels questionnaire to be completed by the participant.

^e^A small number of those who are allocated to the MiQuit group and consent at baseline will be contacted for a qualitative telephone interview to provide feedback on their experience of the study and intervention.

^f^Measured with saliva cotinine and/or exhaled CO levels.

### Visit 1 - baseline

After giving informed consent, participants will be asked to complete a baseline questionnaire, which may be over the telephone with the study team member (*recruitment method one*) or over the telephone with a local RM or in person by the RM (*recruitment method two*). This will confirm contact details and ask about socio- demographic data, smoking attitudes and behaviour (for MiQuit tailoring), heaviness of smoking [15], gestational age, number of births beyond 24 weeks, partner’s (significant other’s) smoking status and health status (EQ-5D) [16].

The researcher will enter the participant details on the electronic database and then randomise the participant. This will ensure the researcher will remain blinded to the participant allocation. The participants are randomised, using the Nottingham Clinical Trials Unit's web-based service, to intervention (MiQuit) or control. For participants in either trial arm, a specific information sheet, which describes only details of procedures employed within that arm, will be generated and sent to them by a non-blinded member of the trial team who is not involved in follow-up. Documentation will also include a leaflet giving some information and advice on stopping smoking, details of a contact point for participants who have questions about trial involvement and details of how participants can reverse their decision to take part (outlined in - removal of participants from intervention or assessments). The PIS, information sheets and consent form will be available in English only, as understanding of English is required to receive the MiQuit intervention.

Follow-up contacts will use a blend of postal, telephone, email/web and SMS text messaging reminders to elicit maximal response rates. For those participants reporting a quit, where possible, a home visit from their local RM will be arranged.

### Visit 2 - 4-week follow-up

*At 4 weeks after randomisation*, participants will be contacted via telephone in order to assess smoking status, numbers of quit attempts lasting more than 24 hours, quality of life information (EQ-5D) and use of NHS and non-NHS smoking cessation support in the period since randomisation. This visit will, in general, be performed via telephone by a member of the study team at the co-ordinating centre, blind to treatment allocation; however, should the study team be unable to contact by the participant by telephone, other methods will be used in an attempt to elicit the best response. These include; posting a short questionnaire with explanatory letter and pre-paid return envelope, or sending a link, which allows web-based completion or by text.

It is possible that if members of the co-ordinating centre study team speak to participants at the 4-week follow-up, they may become un-blinded for the late pregnancy follow-up (visit 3). However, the value of exploring the feasibility of different follow-up approaches for this pilot trial outweighs the small risk of bias presented by this scenario.

### Visit 3 - late pregnancy/postnatal follow-up

*At 36 weeks gestation*: Participants will be asked to complete a questionnaire (data to be collected up to 10 weeks after estimated delivery date will be acceptable for use). This will include quality of life information (EQ-5D) and measures of smoking behaviour, attitudinal/behavioural information, use of NHS smoking cessation support and participants’ ratings of the intervention (MiQuit arm only). As for the 4-week visit, this visit will be performed mainly via telephone by a member of the study team at the co-ordinating centre, blind to treatment allocation. As for the 4-week visit, previously-described alternative methods will be employed to elicit the best response rate.

It is recognised that asking about the intervention at follow-up will result in the researcher who conducts the late pregnancy follow-up becoming un-blinded. To ensure that this has minimal impact on follow-up data obtained, the participants will be asked items about the intervention at the end of the interview/questionnaire starting with the question ‘*Did you receive any stop smoking text messages from the study team*?’

### Visit 4 - validation of smoking status

During this assessment, whether in person or by post, participants will be asked to report their smoking status and recent use of nicotine replacement therapies.

Participants self-reporting abstinence in late pregnancy will have their smoking status validated by exhaled carbon monoxide (CO) readings and/or salivary cotinine samples. The validation of smoking cessation would be initiated as soon as possible after notification of abstinence at visit 3.

Exhaled CO will be measured using a validated hand held CO monitor and can only be taken when a researcher is able to visit the participant in their home. Saliva samples for cotinine (a metabolite of nicotine) analysis could be collected at the same visit. Ideally both forms of assessment would be used, but either CO or cotinine assessment would be sufficient on their own if difficulties in getting a valid sample are experienced with either method. Where it is not possible to visit a participant in their home, a salivary sample pack will be sent by post and participants will be invited to return a sample in a pre-paid postal packet. Written consent agreeing to provide the samples for validation will be obtained from any participants who did not give this consent at baseline (that is, those who were recruited via telephone). This consent will be obtained either during the home visit or by sending a consent form for the participant to complete and return with a postal saliva sample. Cut-off points for biochemical verification will be determined according to the latest evidence, but defined abstinence is likely to be in the region of <9 ppm for CO readings and <10 ng/ml for salivary cotinine [17].

### Duration of the trial

It is anticipated that the total duration of the study will be 20 months. This will include 12 months of recruitment and 8 months of follow-up.

Late pregnancy outcome ascertainment is intended to be at 36 weeks gestation, or at latest, within two weeks of birth. However, if outcome data become available after this point, we will permit it to be used in analyses provided the timing of data collection is no later than 10 weeks after the estimated due date.

Qualitative interviews undertaken as part of a process evaluation will be carried out shortly after the late pregnancy follow-up and are not planned to be carried out any later than 10 weeks after the estimated delivery date.

### End of the trial

The end of the trial will be when the late pregnancy outcome has been ascertained for the final participant or it is too long after this participant’s estimated delivery date (EDD) for such information, if collected, to be used.

### Participant stipends and payments

All participants will receive compensation for their time to the value of £5 for each study visit in the form of a national high street shopping voucher. Those participants who are asked to provide a saliva sample will be sent a £5 voucher with the sample request pack and a second £5 voucher on receipt of a saliva sample. Those participants who participate in a longer in-depth qualitative interview to gain information on their experience of the intervention (MiQuit arm only) will receive £20 in vouchers as compensation for their time.

### Qualitative interviews for process evaluation

A small number of semi-structured telephone interviews will be carried out with a selection of participants from the MiQuit arm in order to address secondary objective 4. This process evaluation will provide important data on participant’s experience of receiving support from MiQuit within the context of their everyday lives and participation in a trial. Semi-structured interviews will enable the researcher to have some control over the data collected to compliment the trial evaluation but at the same time be interactive and allow the participant control also. The findings will greatly assist the design of a definitive trial and optimisation of the intervention.

Participants will be purposively selected according to smoking status and cessation behaviour and use of the MiQuit system, including those who decide to discontinue use of MiQuit. Only participants who have already consented at baseline to be contacted to discuss participation in an interview will be contacted and will receive additional information before the call on what to expect. After completing the late pregnancy follow-up, participants in the MiQuit arm, who indicated at baseline that they were happy to be contacted about participation in an interview, will be sent a specific information sheet and blank consent form. A researcher will call a number of participants (not all) and establish if the participant is still interested in going ahead with the interview. If they are happy to continue, the researcher will request verbal consent for the interview and proceed with the interview. If the participant is interested but unable to continue with the interview at that time, a specific date will be set for the interview to take place. In every case, verbal consent will be recorded by the researcher before the interview goes ahead, and a copy of the consent form will be sent to the participant. The participant will be made aware that the interview will be audio-recorded and that they can withdraw from the interview at any time and that this would not affect their subsequent care or rights. Participants will be recruited until the researchers feel that data saturation has occurred (that is, the point where few novel insights are generated from the most recent interviews). Given the focused nature of the interviews, it is anticipated that this would be reached after interviewing 10 to 15 participants. The interview schedule will include questions on the participants’ general experience of smoking and quitting during pregnancy, their experience of using and interacting with MiQuit, their feelings towards whether and how MiQuit might have helped them engage or maintain a quit attempt, and their thoughts on how it could be improved.

The interviews will be undertaken by experienced personnel and last up to 60 minutes. The interviews will be analysed using framework analysis, assisted by the qualitative analysis software NVivo. This will include looking at two main characteristics of the participants: that is, smoking status and use of system including those who discontinue (text STOP) and how these relate to several aspects of the experience of receiving the intervention including factors that may affect interaction with MiQuit, feelings towards message content and system features and suggestions for improvements for future use.

A number of semi-structured telephone or face to face interviews will also be carried out with a selection of recruiting site research staff involved in the study in order to address secondary objective 5. This will provide important data on the staff experience of running and recruiting into the MiQuit study. As for the participant interviews, the semi-structured interviews will enable the team to have some control over the data collected to complement the trial evaluation but also be interactive to allow the local site research staff to have some control too. The findings will greatly assist the practical design of a definitive trial.

A number of staff from recruiting centres will be selected for interview, to allow a variety of opinions and comments to be collected.

The objectives for these interviews are as follows:

1. To develop a taxonomy for describing MiQuit trial recruitment processes.
2. To describe research midwives’ perceptions of the key facilitators for and barriers against recruitment.
3. To relate key facilitators and barriers to recruitment processes and so further understanding of how, in a definitive trial, recruitment could be maximised.
4. To describe from the perspective of research midwives, those factors, which are perceived to influence acute trusts’ decisions to act as a trial centres (NB: after analysis, we will be in a position to hypothesise about how best to encourage trusts to sign up to being trial centres in a definitive study).

Issues to be covered in staff interviews will include the following:

1. The particular process they used in their site to identify, engage and enrol participants, including the consent process;
2. their views on the local trial processes, including why particular processes were used, what worked well or was challenging, what the study team could have done to make this easier, particularly focussing on issues that it is possible to influence;
3. how decisions are made about Trust involvement in a particular trial and what influenced them to take part, whether they would consider being involved in a similar study again, insights into best way to achieve meaningful collaboration; and
4. their views on the intervention.

The interviews will be audiotaped and analysed using thematic qualitative analysis.

### Statistical methods

#### Sample size and justification

1. *Estimation of key trial parameters*

The primary objective of this pilot trial is to demonstrate the feasibility of running a definitive, multi-centre trial of the MiQuit interevention within an NHS context; with 400 participants, the following key parameters will be estimable within the precision indicated (figures presented are margin of error = half width of 95% confidence interval):

- the recruitment rate of participants from within smokers attending trial centres/hospital to within +/− 1%,
- for individual trial groups, proportion of participants with validated smoking cessation between 4 weeks after randomisation until late pregnancy to within +/− 4%, and
- for both trial groups, the combined proportion of participants who quit in late pregnancy (cessation as defined above) to within +/− 3%, assuming that the actual proportion of participants quitting is around 10%.

2. *Estimate for short-term efficacy*

A secondary objective of this pilot trial is to estimate the effectiveness of the MiQuit intervention for promoting smoking cessation. Providing evidence to support the notion that this intervention is likely to be effective would increase the research team’s future chances of obtaining funding to conduct a definitive trial. We will investigate efficacy of MiQuit at 4 weeks after randomisation by comparing self-reported, 7-day, point-prevalence, cessation rates between trial groups. It should be noted that this is a surrogate end point for that which we plan to use in any definitive trial; a potential strength of using this is that event rates (that is, proportion of participants achieving cessation) are likely to be higher at this time point, which is closer to randomisation than the late pregnancy-assessment point, and this could provide greater power to detect between group differences. A potential disadvantage is that the relationship between this surrogate endpoint and cessation in late pregnancy (approx 36 weeks) is not known; consequently, findings using data obtained at this time point should not be used in isolation to assess whether or not a definitive trial should be conducted. Consequently, using 95% and 80% confidence intervals (CIs), Tables 2 and 3 illustrate for this trial with 400 participants, the precision for detecting different ORs representing differences between groups over a range of control group event (that is, quit) rates. The choices of parameters for the tables are explained below.

**Table 2:** **Estimates for treatment effect: precision estimates presented as 95% confidence intervals**

***95% CI Odds ratio***	**10% control event rate N = 200 in each group**	**15% control event rate N = 200 in each group**	**20% control event rate N = 200 in each group**
1.3	0.70 to 2.42	0.77 to 2.20	0.81 to 2.09
1.5	0.82 to 2.76	0.90 to 2.51	0.94 to 2.39
1.8	1.0 to 3.26	1.09 to 2.98	1.14 to 2.85

**Table 3:** **Estimates for treatment effect: precision estimates presented as 80% confidence intervals**

***80% CI Odds ratio***	**10% control event rate N = 200 in each group**	**15% control event rate N = 200 in each group**	**20% control event rate N = 200 in each group**
1.3	0.87 to 1.95	0.92 to 1.83	0.95 to 1.77
1.5	1.01 to 2.23	1.07 to 2.10	1.11 to 2.03
1.8	1.22 to 2.65	1.29 to 2.51	1.33 to 2.43

*Control group event (quit) rates*: There are few data with which we can estimate control group event (that is, proportion of participants achieving cessation) rates at 1 month after randomisation, but accurate estimates are important to inform meaningful sample size estimates. One recent trial (MiQuit feasibility trial), tested a very similar version of this trial’s intervention, but in this study 7-day point-prevalence abstinence was measured in similar manner as we propose at *12 weeks* after randomisation, rather than at 1 month after; in this trial, the control group quit rate was 19.6% [11]. Two recent UK trials have enrolled pregnant smokers, albeit with different characteristics, and have measured cessation outcomes at 1 month after randomisation. A trial of nicotine patches found a 14% control group rate of self-reported, *prolonged* cessation between randomisation and 1 month [18], and control group women recruited to an exercise intervention RCT had a 13.7% validated abstinence rate, again for reported, *prolonged* abstinence during the same period (unpublished findings) [19]. Overall, therefore, assuming that control group quit rates will fall within in a 10 to 20% range seems reasonable.

3. *Estimates for treatment effect*

MiQuit is a self-help intervention and a systematic review of self-help interventions used for smoking cessation in pregnancy found a pooled estimate for the effectiveness of these, which corresponds to an Odds Ratio (OR) and (95% CI) in favour of using such interventions of 1.83 (1.23 to 2.73) [7]. Using data obtained at 3 months after randomisation, the MiQuit feasibility trial found an OR in favour of using MiQuit of 1.68 (0.66 to 4.31) [11]. Consequently, using a range of OR from 1.3 to 1.8 is appropriate.

*Estimating cessation in late pregnancy:* For modelling analyses, we will use a trial-derived estimate for the treatment effect of the intervention derived from between-group differences in validated, longer term quit rates, measured in late pregnancy (that is, at approximately 36 weeks gestation). Again deriving this estimate is a secondary objective of the trial. Tables 4 and 5 use 95% and 80% confidence intervals (CIs), to illustrate for this trial with 400 participants, the precision for detecting different ORs representing differences between groups over a range of control group event (that is, proportion of participants achieving cessation) rates. The proportion of participants in the control group who quit is based on 5% and 10%, as this reflects findings in the three trials described above [11,18,19].

**Table 4:** **Estimating cessation in late pregnancy: precision estimates presented as 95% confidence intervals**

***95% CI Odds ratio***	**5% control event rate N = 200 in each group**	**8% control event rate N = 200 in each group**	**10% control event rate N = 200 in each group**
1.3	0.56 to 3.05	0.65 to 2.58	0.70 to 2.42
1.5	0.66 to 3.44	0.77 to 2.93	0.82 to 2.76
1.8	0.81 to 4.02	0.94 to 3.46	1.0 to 3.26

**Table 5:** **Estimating cessation in late pregnancy: precision estimates presented as 80% confidence intervals**

***80% CI Odds ratio***	**5% control event rate N = 200 in each group**	**8% control event rate N = 200 in each group**	**10% control event rate N = 200 in each group**
1.3	0.75 to 2.27	0.83 to 2.04	0.87 to 1.95
1.5	0.87 to 2.58	0.97 to 2.33	1.01 to 2.23
1.8	1.06 to 3.05	1.18 to 2.76	1.22 to 2.65

### Data analysis

Members of the research team, led by the study statistician, will undertake the analyses. A full statistical analysis plan will be finalised and agreed by the Trial Steering Committee prior to data analysis. Initially, participants in the two intervention groups will be described separately with respect to the following:

1. Centre
2. Age at randomisation
3. Ethnic group (white, other)
4. Gestation at randomisation (<16 weeks, 16+ weeks)
5. Heaviness of smoking
6. Number of births beyond 24 weeks,
7. Previous history of prenatal smoking
8. Partner/significant other’s smoking status
9. Participant’s health status (EQ5D)
10. Participant’s motivation to quit

Analyses will primarily address the feasibility objectives of the study. We will determine the recruitment rate, with 95% confidence intervals, of participants who take part in the study from pregnant smokers attending trial centre. For each trial treatment group, we will estimate the proportions of participants, with 95% confidence intervals, achieving validated smoking cessation between 4 weeks after randomisation until late pregnancy (at approximately 36 weeks gestation). We will also estimate the proportion of participants, with 95% confidence intervals, who achieve validated smoking cessation in late pregnancy (approximately at 36 weeks gestation) for both trial groups combined.

We will perform chi-squared tests to assess the association for the proportion of participants who achieve 4-week and late-pregnancy smoking outcomes between the trial treatment groups. Logistic regression models will then be used to compare the outcomes between the two trial groups with adjustment for factors used to stratify the randomisation (centre and gestation at randomisation; <16 weeks versus ≥16 weeks) through including these as fixed covariates in the model. These analyses will be pragmatic and based on intention-to-treat. Additionally, we will create the following graphs to look at the pattern of the outcome response across centres:

1. Average outcome in the intervention group versus the average outcome in the control group per centre.
2. Average difference in outcome between the intervention and control groups per centre versus centre.

Further multiple logistic regression analyses will be conducted to assess the strength of association between baseline covariates and 4-week and late-pregnancy smoking outcomes, which will include the following:

1. Heaviness of smoking.
2. Partner/significant other’s smoking status.
3. Education as a measure of SES.

These variables have been chosen as potentially requiring adjustment because, in previous studies, they have been shown to be associated with smoking cessation in pregnancy [20,21].

Other smoking outcomes will be analysed using similar methods: 95% confidence intervals will be presented along with exact two-sided *P* values. Descriptive analyses will be performed to assess the effect of the intervention on the use of NHS cessation support, use of interactive features of the intervention and social cognitive variables.

We also plan to explore the mechanism of effect for the MiQuit intervention that is, how the intervention might change smoking behaviour. For this, we will undertake mediation analyses to explore whether potential changes in social cognitive determinants of smoking cessation explain changes in quitting behaviour. This will be a secondary analysis and, therefore, covered in a separate paper rather than in the main trial outcomes paper.

Health economic analysis will investigate the potential incremental cost-effectiveness of MiQuit using costs (actual and estimated) of delivering the intervention and estimates for its effectiveness derived from the trial. Models developed in previous studies will be used to project the likely cessation rates into longer term estimates of life years gained.

### Assessment of efficacy

The smoking outcome measure used to estimate efficacy will be self-reported, prolonged abstinence from smoking from 4 weeks after randomisation until late pregnancy, validated by exhaled CO and/or saliva cotinine estimation. Smoking up to five cigarettes during the abstinence period will be permitted and consistent with a positive outcome.

### Assessment of safety

As this evaluation concerns a behavioural intervention we are not assessing any safety variables or collecting any adverse events.

### Procedures for missing, unused and spurious data

Our primary analyses will use an intention-to-treat approach, analysing all participants’ outcome data within the intervention group to which they were randomised, assuming, as is consistent with the Russell Standard, that participants with missing smoking status data are smoking [13]. Participants are advised to withdraw from the study if they have a miscarriage or still birth or where the infant has died following birth; however, if they do not and this is established at follow-up they may decline to provide information. These women will be included in the analyses and assumed to be smoking.

For the descriptive analyses, where there are missing baseline socio-demographic data, a separate category will be created and reported to represent the missing data. For statistical analyses involving socio-demographic variables with missing data, if the proportion of missing data is <5% and roughly similar in both intervention groups, then we will explore the robustness of using a complete-case analysis and an analysis including allowing for the missing data through analysing missing data as a separate category. If >5% of socio-demographic data are missing, then we will consider using imputation methods, such as multiple imputations. The impact of the imputations methods will be assessed fully in sensitivity analyses.

### Definition of populations analysed

For the efficacy and cost-effectiveness estimate assessments, all randomised participants will be used in the analyses using intention-to-treat population as defined below. All participants in the intention-to-treat population will be used for establishing use of NHS smoking cessation support and for the mechanism of effect analysis.

### Intention-to-treat population

The intention-to-treat (ITT) population is defined for this trial as all pregnant smokers who were randomised into the trial. Participants will be analysed based on the intervention group to which they were randomised. For participants with missing smoking-related outcomes, we will use the Russell Standard, where these participants will be assumed to be smoking [13].

### Sensitivity analyses

Two populations will be used for the sensitivity analyses on the 4-week and late-pregnancy smoking outcome measures:

1. All pregnant smokers who were randomised into the trial, and who have outcome data available for analysis (complete case analysis). Participants will be analysed based on the intervention group to which they were randomised.
2. All pregnant smokers who were randomised into the trial, and who, in the intervention group, self-reported at follow up that they received the intervention leaflet and text messages (per protocol analysis).

### Health economics

Per-participant costs of providing the MiQuit service will be calculated. and EQ-5D data will be used with this to estimate the incremental cost-effectiveness of offering the MiQuit intervention. Given that this is a pilot study rather than a definitive trial, it is acknowledged that the estimate for incremental cost-effectiveness will have limited accuracy and so a range of different potential values will be used to model the potential lifetime cost effectiveness of introducing MiQuit into routine clinical practice. Modelling will use i) estimates for uptake of NHS services by smokers and non-smokers after pregnancy, which have been derived from a similar recent trial that employed post-partum follow up [18], and ii) a model, which values smoking cessation in pregnancy in economic terms and is currently being developed by a PhD student.

### Data management

Data will be entered electronically on a trial specific database. Only research midwives and the research study team will have database access, which permits them to make new entries and also to access relevant personal data collected from participants at their sites (for example, contact details).

To enable anonymisation, each participant will be assigned a unique trial identity code number, allocated at randomisation, for use on all trial documents and the electronic database.

The database will be maintained on a server located within the Nottingham Clinical Trials Unit, University of Nottingham (Chief Investigator’s institution). The database has a regular back-up routine, and will be password protected.

Anonymised data, which have been sent to MiQuit and which have been used to individualise SMS text messages, will be held on a secure server within the Institute of Public Health at the University of Cambridge. Only authorised trial staff shall have access to trial documentation other than for the regulatory requirements.

All trial staff and investigators will endeavour to protect the rights of the trial’s participants to privacy and informed consent and will adhere to the Data Protection Act, 1998.

Electronic data will be backed up every 24 hours to both local and remote media in encrypted format. Electronic audio files of the interviews will be stored in an encrypted format. Transcribed interviews will be anonymised.

Monitoring of trial data shall include confirmation of informed consent; source data verification; data storage and data transfer procedures; local quality control checks and procedures, back-up and disaster recovery of any local databases and validation of data manipulation. The Trial Manager, or where required, a nominated designee of the Sponsor, shall carry out monitoring of trial data as an on-going activity.

All records and documents regarding the conduct of the study will be retained for at least 7 years or for longer if required. The trial master file and trial documents held by the Chief Investigator on behalf of the sponsor will be archived at secure archive facilities at the University of Nottingham. This archive shall include all trial databases and associated meta-data encryption codes.

### Transport, storage and analysis of saliva samples

The use of oral fluid samples (saliva) is a non-invasive way of measuring cotinine, a nicotine metabolite [22]. Saliva samples will be collected at visit 4 (around 36 weeks gestation) to verify smoking status by a researcher or by post if a visit is not possible. Saliva samples will be obtained using clean salivettes; it will involve the participant placing a sterile swab under the tongue, for up to 5 minutes until it is moist and then placing the swab into a sterile vessel. All samples will be stored in a linked anonymised format in a secure freezer storage unit (−20°C) within Nottingham Health Science Biobank (NHSB) according to their approved protocols. Where researchers obtain samples by visiting participants who live at a distance that would make it impractical to deliver these to Nottingham in person, these samples will be posted by the study team in suitable packaging, and when received, the study team will transfer them to NHSB. Samples are stable at ambient temperatures for several days. For postal samples, the participant will be asked to state the date they provided the sample on the label. Participants will be advised to return the sample (using the protective pre-paid and addressed packaging) on the day they provide the sample. Again these will be transferred to the NHSB by a member of the study team. Nottingham Health Science Biobank has been given full approval by the HTA to be a full licence holder, meeting all legislation requirements.

Once all samples have been collected for the study, NHSB will arrange transportation of samples by courier to ABS Laboratories Ltd, Hertfordshire for analysis in a single batch shipment. The shipment will contain a complete inventory of all samples, along with the name of the person responsible for sending the samples. The master database to link all samples will be held by the Nottingham study team in a password encrypted file. The laboratory will quantify salivary cotinine levels using a quantitative enzyme immunoassay technique (EIA). Once the analysis has been completed the saliva samples will be destroyed in accordance with the Human Tissue Act 2004, this will only occur once the study team have received the results and have analysed the data to ensure that all samples remain in a normal range and do not require retesting.

### Dissemination

Results will be written up for publication in peer reviewed journals and disseminated at local, national, and international meetings where appropriate. A lay summary will be produced and distributed to those participants who have indicated they would like to receive a copy and other interested parties.

Participants will not be identified in any publications or presentations resulting from this study.

### User and public involvement

A Public and Patient Involvement representative has contributed to this protocol, to trial documents and to the development of the intervention. In addition, several individuals who took part in a pilot trial of MiQuit have been consulted and have provided guidance on the refinement and delivery of MiQuit.

### Indemnity

Insurance and indemnity for trial participants and trial staff is covered within the NHS Indemnity Arrangements for clinical negligence claims in the NHS, issued under cover of HSG (96)48.

The University of Nottingham as research Sponsor indemnifies its staff, research participants and research protocols with both public liability insurance and clinical trials insurance. These policies include provision for indemnity in the event of a successful litigious claim for proven non-negligent harm.

### Trial management

The Chief Investigator has overall responsibility for the study and will oversee all study management. The Trial Management Group (TMG) will be responsible for the day to day running of the trial. The TMG will be supported by and report to an independent Trial Steering Committee. Trial co-ordination will be managed centrally by the study team based in Nottingham led by a trial manager and guided by the TMG. The Cambridge research team will manage a server hosting the MiQuit intervention; however, the Chief Investigator (Nottingham) will be custodian of all study data.

### Ethics approval

Ethical approval for this study was granted by Nottingham 1 Research Ethics Committee (NRES reference 13/EM/0427).

### Sponsor

The trial is sponsored by the University of Nottingham.

## Discussion

Once completed, this pilot trial will provide the necessary data to plan for larger definitive study to investigate whether or not self-help cessation support [7] provided in an interactive text message format can help pregnant women to achieve cessation. This pilot trial provides information, which complements that from our earlier feasibility trial [11] and has been necessary for a number of reasons.

First, the primary outcome for the earlier study was self-reported, 7-day, point-prevalence, smoking cessation [12] measured at 12 weeks after trial entry [11]. Whilst any period of smoking cessation is likely to be beneficial for pregnant women and their infants, maximal health gain for infants is likely to occur if mothers maintain abstinence throughout pregnancy. Additionally, it is possible that women who remain smoke free for the whole of pregnancy will be better able to avoid relapse back to smoking after childbirth and, for both reasons, a definitive study should aim to detect any impact on women’s smoking throughout the whole of pregnancy. Most smoking cessation trials encourage participants to set a quit date and cessation outcomes are measured prospectively from this point; however, MiQuit is a ‘cessation-induction’ intervention, which encourages, but does not require users to set a definite quit date. Finding appropriate time points in pregnancy from which continuous cessation can be measured, monitored and validated, after participants have been exposed to MiQuit, is an important issue that the pilot trial is attempting to resolve. Achieving high ascertainment rates for piloted methods of measuring continuous cessation will mean that our piloted outcome could be used to measure and validate smoking cessation throughout the whole of pregnancy in any future definitive RCT.

Second, the treatment effects attributable to smoking cessation interventions are generally inversely proportional to their intensity [23] and MiQuit is a low-intensity intervention. For a definitive study to have sufficient power to show whether or not MiQuit has efficacy for smoking cessation in pregnancy, such a study could need a sample size of over 3,000 participants (estimated using data from previous MiQuit trial [11]). Clearly, to plan a definitive study that combines a sufficiently high chance of providing a definitive answer with efficient use of resources, the best possible information is needed. This pilot trial will provide the first estimate for the treatment effect that MiQuit might have for influencing continuous smoking cessation between earlier and later (approximately 36 weeks) pregnancy; although the pilot is not powered to provide such an estimate with high precision, it will nevertheless, be a very valuable parameter. Additionally, knowing the combined treatment groups’ cessation rates at 36 weeks will also be useful to inform definitive-trial sample size calculations.

Third, recruitment rates from this study will be vital for assessing the feasibility of delivering a large definitive RCT investigating smoking cessation in pregnancy and using English National Health Service (NHS) research structures. Since 2006, the English NHS has developed and revised research networks, funded from the health service budget and orientated towards delivering clinical research; there is a particular focus on RCTs that influence clinical practice [24]. Networks provide staff working across the NHS whose remit is to recruit to research studies and researchers who successfully access network staff support can efficiently deliver multi-centre RCTs. However, negotiating access to research networks and subsequently finding the most efficient ways of combining their efforts with those of a university-based research team can be challenging and, significantly the two most recent English pregnancy smoking cessation trials [18,19] were completed with relatively little research network input. To date, overall pilot trial recruitment has been strong and it appears that study methods could, feasibly be used to recruit to a larger definitive study. However, across the 16 trial centres from which this pilot recruits, there has been marked variation in recruitment rates. Consequently, lessons learned from our planned pilot trial process evaluation should help us to better understand the reasons for different recruitment rates in different centres and, potentially to enhance the research network staff experience of recruiting to the trial for any future study.

Given the nature of this pilot trial, discussion has principally focussed on methodological issues. However, if after this study, a definitive RCT is conducted and MiQuit is found to be effective for smoking cessation in pregnancy, this very cheap and likely (in that circumstance) highly cost-effective intervention could easily be made very widely available in many different ways to support smoking cessation in pregnancy.

## Trial status

Recruitment began in February 2014, with the first participant randomised on 18 February 2014. At the time of the manuscript submission, the trial was still recruiting with 385 recruited by 27 August 2014. This article is based on protocol version 2.0, 14 May 2014.

## Abbreviations

CLRN: Comprehensive Local Research Network
CO: carbon monoxide
EDD: estimated delivery date
EQ-5D: EuroQol-5D-3 Level Questionnaire
NHS: National Health Service
NHSB: Nottingham Health Science Biobank
PIS: participant information sheet
RCT: randomised controlled trial
RM: research midwife
TMG: trial management group
